# Di-μ-chlorido-bis­(chlorido{2-[(4-ethyl­phen­yl)imino­meth­yl]pyridine-κ^2^ *N*,*N*′}copper(II))

**DOI:** 10.1107/S1600536811032053

**Published:** 2011-08-27

**Authors:** Saeed Dehghanpour, Ali Mahmoudi, Mehdi Khalaj, Somayeh Abbasi, Fresia Mojahed

**Affiliations:** a Department of Chemistry, Alzahra University, Tehran, Iran; b Department of Chemistry, Islamic Azad University, Karaj Branch, Karaj, Iran; c Department of Chemistry, Islamic Azad University, Buinzahra Branch, Qazvin, Iran; d Department of Chemistry, Faculty of Science, Urmia University, Urmia, 57159-165, Iran

## Abstract

The binuclear title complex, [Cu_2_Cl_4_(C_14_H_14_N_2_)_2_], is located on a crystallographic inversion centre. The Cu^II^ ion is in a distorted square-pyramid coordination environment formed by the bichelating *N*-heterocyclic ligand, two bridging Cl atoms and one terminal Cl atom. One of the bridging Cu—Cl bonds is significantly longer than the other.

## Related literature

For the synthesis of the ligand, see: Dehghanpour *et al.* (2009 ▶). For background to diimine complexes and related structures, see: Mahmoudi *et al.* (2009 ▶); Salehzadeh *et al.* (2011 ▶).
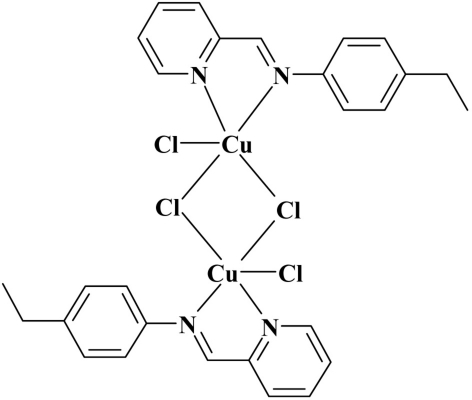


## Experimental

### 

#### Crystal data

[Cu_2_Cl_4_(C_14_H_14_N_2_)_2_]
*M* *_r_* = 689.42
Monoclinic, 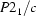

*a* = 10.1254 (3) Å
*b* = 8.8384 (3) Å
*c* = 16.2117 (4) Å
β = 100.8830 (18)°
*V* = 1424.73 (7) Å^3^
*Z* = 2
Mo *K*α radiation
μ = 1.89 mm^−1^
*T* = 150 K
0.18 × 0.18 × 0.12 mm

#### Data collection

Nonius KappaCCD diffractometer
Absorption correction: multi-scan (*SORTAV*; Blessing, 1995 ▶) *T*_min_ = 0.672, *T*_max_ = 0.795
13286 measured reflections
3246 independent reflections
2568 reflections with *I* > 2σ(*I*)
*R*_int_ = 0.043

#### Refinement

*R*[*F* ^2^ > 2σ(*F* ^2^)] = 0.037
*wR*(*F* ^2^) = 0.090
*S* = 1.11
3246 reflections
173 parameters
H-atom parameters constrained
Δρ_max_ = 0.60 e Å^−3^
Δρ_min_ = −0.59 e Å^−3^

### 

Data collection: *COLLECT* (Nonius, 2002 ▶); cell refinement: *DENZO-SMN* (Otwinowski & Minor, 1997 ▶); data reduction: *DENZO-SMN*; program(s) used to solve structure: *SIR92* (Altomare *et al.*, 1994 ▶); program(s) used to refine structure: *SHELXTL* (Sheldrick, 2008 ▶); molecular graphics: *PLATON* (Spek, 2009 ▶); software used to prepare material for publication: *SHELXTL*.

## Supplementary Material

Crystal structure: contains datablock(s) I, global. DOI: 10.1107/S1600536811032053/kj2183sup1.cif

Structure factors: contains datablock(s) I. DOI: 10.1107/S1600536811032053/kj2183Isup2.hkl

Additional supplementary materials:  crystallographic information; 3D view; checkCIF report

## Figures and Tables

**Table 1:** Selected bond lengths (Å)

Cu1—N1	2.040 (2)
Cu1—N2	2.046 (2)
Cu1—Cl2	2.2423 (7)
Cu1—Cl1	2.3067 (7)
Cu1—Cl1^i^	2.5883 (7)

Symmetry code: (i) 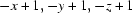.

**Fig. 1.**
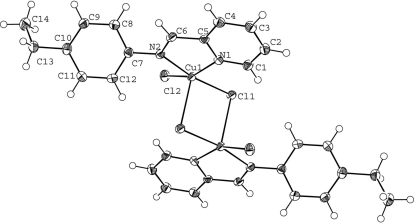
A view of the structure of the title complex, with displacement ellipsoids drawn at 50% probability level.

## supplementary crystallographic information

### Comment

In our ongoing studies on the synthesis, structural and spectroscopic characterization of transition metal complexes with diimine ligands (Dehghanpour *et al.*, 2009; Salehzadeh *et al.*, 2011), here we report the crystal structure of the title complex. The title complex was prepared by the reaction of CuCl_2_ with the bidentate ligand (4-methylphenyl)pyridin-2-ylmethyleneamine (Mahmoudi *et al.*, 2009).

The molecluar structure of the title complex is shown in Fig. 1. The Cu^II^ ion is in a distorted squar pyramid environment formed by a bis-chelating ligand, two bridging Cl atoms and one terminal Cl atom. A comparison of the dihedral angles between the planes of the pyridine, chelate and the benzene ring indicate that the ligand is distorted from planarity, with twist of 41.9 (2)° between the chelate (N1C5C6N2) and the benzene (C7C8C9C10C11C12) planes.One of the bridging Cu—Cl bonds is significantly longer than the other.

### Experimental

The title complex was prepared by the reaction of CuCl_2_ (13.4 mg, 0.1 mmole) and (4-methylphenyl)pyridin-2-ylmethyleneamine (21.0 mg, 0.1) in 15 ml methanol at room temperature. The solution was allowed to stand at room temperature and green crystals of the title compound suitable for X-ray analysis precipitated within few days.

### Refinement

H atoms bonded to C atoms were placed in calculated positions with C-H = 0.95Å and included in the refinement in a riding-model approximation with U_iso_(H) = 1.5U_eq_(C) for methyl H and U_iso_(H) = 1.2U_eq_(C) for other C-H.

### Crystal data

**Table:**

[Cu_2_Cl_4_(C_14_H_14_N_2_)_2_]	*F*(000) = 700
*M**_r_* = 689.42	*D*_x_ = 1.607 Mg m^−^^3^
Monoclinic, *P*2_1_/*c*	Mo *K*α radiation, λ = 0.71073 Å
Hall symbol: -P 2ybc	Cell parameters from 5841 reflections
*a* = 10.1254 (3) Å	θ = 2.6–27.5°
*b* = 8.8384 (3) Å	µ = 1.89 mm^−^^1^
*c* = 16.2117 (4) Å	*T* = 150 K
β = 100.8830 (18)°	Block, green
*V* = 1424.73 (7) Å^3^	0.18 × 0.18 × 0.12 mm
*Z* = 2	

### Data collection

**Table:**

Nonius KappaCCD diffractometer	3246 independent reflections
Radiation source: fine-focus sealed tube	2568 reflections with *I* > 2σ(*I*)
graphite	*R*_int_ = 0.043
Detector resolution: 9 pixels mm^-1^	θ_max_ = 27.5°, θ_min_ = 2.6°
φ scans and ω scans with κ offsets	*h* = −13→10
Absorption correction: multi-scan (*SORTAV* (Blessing, 1995)	*k* = −11→11
*T*_min_ = 0.672, *T*_max_ = 0.795	*l* = −20→21
13286 measured reflections	

### Refinement

**Table:**

Refinement on *F*^2^	Primary atom site location: structure-invariant direct methods
Least-squares matrix: full	Secondary atom site location: difference Fourier map
*R*[*F*^2^ > 2σ(*F*^2^)] = 0.037	Hydrogen site location: inferred from neighbouring sites
*wR*(*F*^2^) = 0.090	H-atom parameters constrained
*S* = 1.11	*w* = 1/[σ^2^(*F*_o_^2^) + (0.0322*P*)^2^ + 1.7627*P*] where *P* = (*F*_o_^2^ + 2*F*_c_^2^)/3
3246 reflections	(Δ/σ)_max_ = 0.001
173 parameters	Δρ_max_ = 0.60 e Å^−^^3^
0 restraints	Δρ_min_ = −0.59 e Å^−^^3^

### Special details

**Table:**

Experimental. multi-scan from symmetry-related measurements SORTAV (Blessing, 1995)
Geometry. All e.s.d.'s (except the e.s.d. in the dihedral angle between two l.s. planes) are estimated using the full covariance matrix. The cell e.s.d.'s are taken into account individually in the estimation of e.s.d.'s in distances, angles and torsion angles; correlations between e.s.d.'s in cell parameters are only used when they are defined by crystal symmetry. An approximate (isotropic) treatment of cell e.s.d.'s is used for estimating e.s.d.'s involving l.s. planes.
Refinement. Refinement of *F*^2^ against ALL reflections. The weighted *R*-factor *wR* and goodness of fit *S* are based on *F*^2^, conventional *R*-factors *R* are based on *F*, with *F* set to zero for negative *F*^2^. The threshold expression of *F*^2^ > σ(*F*^2^) is used only for calculating *R*-factors(gt) *etc*. and is not relevant to the choice of reflections for refinement. *R*-factors based on *F*^2^ are statistically about twice as large as those based on *F*, and *R*- factors based on ALL data will be even larger.

### Fractional atomic coordinates and isotropic or equivalent isotropic displacement parameters (Å^2^)

**Table:**

	*x*	*y*	*z*	*U*_iso_*/*U*_eq_	
Cu1	0.49287 (3)	0.40798 (4)	0.59239 (2)	0.01724 (11)	
Cl1	0.65548 (6)	0.56294 (8)	0.55534 (4)	0.01911 (16)	
Cl2	0.41609 (7)	0.59028 (8)	0.66720 (4)	0.02404 (17)	
N1	0.5981 (2)	0.2194 (3)	0.57146 (14)	0.0185 (5)	
N2	0.3756 (2)	0.2450 (3)	0.63198 (13)	0.0178 (5)	
C1	0.7060 (3)	0.2085 (3)	0.53486 (17)	0.0222 (6)	
H1A	0.7364	0.2955	0.5095	0.027*	
C2	0.7742 (3)	0.0725 (3)	0.53322 (18)	0.0240 (6)	
H2A	0.8512	0.0678	0.5076	0.029*	
C3	0.7307 (3)	−0.0556 (3)	0.56855 (18)	0.0243 (6)	
H3A	0.7777	−0.1486	0.5685	0.029*	
C4	0.6167 (3)	−0.0455 (3)	0.60428 (18)	0.0224 (6)	
H4A	0.5825	−0.1322	0.6279	0.027*	
C5	0.5544 (3)	0.0924 (3)	0.60479 (16)	0.0173 (6)	
C6	0.4297 (3)	0.1137 (3)	0.63732 (17)	0.0197 (6)	
H6A	0.3902	0.0318	0.6618	0.024*	
C7	0.2512 (3)	0.2625 (3)	0.66176 (17)	0.0193 (6)	
C8	0.2360 (3)	0.1939 (3)	0.73601 (17)	0.0226 (6)	
H8A	0.3085	0.1399	0.7688	0.027*	
C9	0.1134 (3)	0.2047 (3)	0.76209 (17)	0.0225 (6)	
H9A	0.1029	0.1566	0.8129	0.027*	
C10	0.0059 (3)	0.2836 (3)	0.71632 (18)	0.0228 (6)	
C11	0.0253 (3)	0.3551 (4)	0.64241 (18)	0.0250 (6)	
H11A	−0.0465	0.4107	0.6100	0.030*	
C12	0.1477 (3)	0.3464 (3)	0.61556 (17)	0.0233 (6)	
H12A	0.1600	0.3974	0.5660	0.028*	
C13	−0.1281 (3)	0.2884 (4)	0.74484 (18)	0.0270 (7)	
H13A	−0.1718	0.1881	0.7348	0.032*	
H13B	−0.1868	0.3633	0.7103	0.032*	
C14	−0.1168 (3)	0.3292 (4)	0.83719 (19)	0.0332 (7)	
H14A	−0.2069	0.3336	0.8510	0.050*	
H14B	−0.0731	0.4280	0.8479	0.050*	
H14C	−0.0633	0.2523	0.8721	0.050*	

### Atomic displacement parameters (Å^2^)

**Table:**

	*U*^11^	*U*^22^	*U*^33^	*U*^12^	*U*^13^	*U*^23^
Cu1	0.01785 (18)	0.0172 (2)	0.01712 (18)	−0.00025 (13)	0.00446 (13)	0.00089 (13)
Cl1	0.0199 (3)	0.0202 (4)	0.0171 (3)	−0.0028 (3)	0.0029 (2)	0.0011 (3)
Cl2	0.0224 (3)	0.0254 (4)	0.0252 (4)	0.0003 (3)	0.0070 (3)	−0.0061 (3)
N1	0.0169 (11)	0.0186 (12)	0.0195 (12)	0.0009 (9)	0.0022 (9)	0.0004 (9)
N2	0.0186 (11)	0.0191 (12)	0.0158 (11)	−0.0023 (9)	0.0037 (9)	−0.0004 (9)
C1	0.0219 (14)	0.0231 (16)	0.0222 (14)	−0.0025 (12)	0.0053 (11)	0.0021 (12)
C2	0.0212 (14)	0.0249 (16)	0.0269 (16)	0.0016 (12)	0.0065 (12)	−0.0005 (12)
C3	0.0254 (15)	0.0213 (16)	0.0259 (16)	0.0030 (12)	0.0044 (12)	−0.0015 (12)
C4	0.0245 (15)	0.0173 (14)	0.0245 (15)	0.0002 (12)	0.0021 (12)	0.0013 (12)
C5	0.0182 (13)	0.0201 (15)	0.0128 (13)	−0.0013 (11)	0.0008 (10)	0.0013 (11)
C6	0.0222 (14)	0.0201 (15)	0.0168 (14)	−0.0042 (11)	0.0036 (11)	0.0022 (11)
C7	0.0182 (13)	0.0203 (15)	0.0194 (14)	−0.0024 (11)	0.0038 (11)	−0.0011 (11)
C8	0.0225 (14)	0.0237 (15)	0.0211 (14)	0.0015 (12)	0.0032 (11)	0.0017 (12)
C9	0.0238 (14)	0.0254 (16)	0.0190 (14)	−0.0008 (12)	0.0057 (11)	0.0012 (12)
C10	0.0207 (14)	0.0230 (16)	0.0253 (15)	−0.0046 (12)	0.0062 (12)	−0.0042 (12)
C11	0.0192 (14)	0.0306 (17)	0.0246 (15)	0.0020 (12)	0.0024 (11)	0.0027 (13)
C12	0.0252 (15)	0.0265 (16)	0.0181 (14)	0.0014 (12)	0.0037 (11)	0.0045 (12)
C13	0.0208 (14)	0.0346 (18)	0.0262 (15)	−0.0013 (13)	0.0061 (12)	0.0001 (13)
C14	0.0292 (16)	0.043 (2)	0.0295 (17)	0.0026 (15)	0.0116 (14)	−0.0006 (15)

### Geometric parameters (Å, °)

**Table:**

Cu1—N1	2.040 (2)	C6—H6A	0.9500
Cu1—N2	2.046 (2)	C7—C8	1.382 (4)
Cu1—Cl2	2.2423 (7)	C7—C12	1.383 (4)
Cu1—Cl1	2.3067 (7)	C8—C9	1.388 (4)
Cu1—Cl1^i^	2.5883 (7)	C8—H8A	0.9500
Cl1—Cu1^i^	2.5883 (7)	C9—C10	1.384 (4)
N1—C1	1.342 (3)	C9—H9A	0.9500
N1—C5	1.356 (3)	C10—C11	1.401 (4)
N2—C6	1.280 (3)	C10—C13	1.514 (4)
N2—C7	1.439 (3)	C11—C12	1.390 (4)
C1—C2	1.388 (4)	C11—H11A	0.9500
C1—H1A	0.9500	C12—H12A	0.9500
C2—C3	1.378 (4)	C13—C14	1.523 (4)
C2—H2A	0.9500	C13—H13A	0.9900
C3—C4	1.389 (4)	C13—H13B	0.9900
C3—H3A	0.9500	C14—H14A	0.9800
C4—C5	1.373 (4)	C14—H14B	0.9800
C4—H4A	0.9500	C14—H14C	0.9800
C5—C6	1.469 (4)		
			
N1—Cu1—N2	80.19 (9)	N2—C6—H6A	120.7
N1—Cu1—Cl2	157.23 (7)	C5—C6—H6A	120.7
N2—Cu1—Cl2	93.15 (7)	C8—C7—C12	120.6 (2)
N1—Cu1—Cl1	91.19 (6)	C8—C7—N2	119.6 (2)
N2—Cu1—Cl1	170.06 (7)	C12—C7—N2	119.8 (2)
Cl2—Cu1—Cl1	92.96 (3)	C7—C8—C9	119.2 (3)
N1—Cu1—Cl1^i^	99.05 (6)	C7—C8—H8A	120.4
N2—Cu1—Cl1^i^	95.18 (6)	C9—C8—H8A	120.4
Cl2—Cu1—Cl1^i^	103.25 (3)	C10—C9—C8	122.0 (3)
Cl1—Cu1—Cl1^i^	91.04 (2)	C10—C9—H9A	119.0
Cu1—Cl1—Cu1^i^	88.96 (2)	C8—C9—H9A	119.0
C1—N1—C5	118.1 (2)	C9—C10—C11	117.5 (3)
C1—N1—Cu1	128.83 (19)	C9—C10—C13	120.6 (3)
C5—N1—Cu1	112.96 (17)	C11—C10—C13	121.8 (3)
C6—N2—C7	117.7 (2)	C12—C11—C10	121.3 (3)
C6—N2—Cu1	113.14 (18)	C12—C11—H11A	119.3
C7—N2—Cu1	128.77 (18)	C10—C11—H11A	119.3
N1—C1—C2	121.4 (3)	C7—C12—C11	119.3 (3)
N1—C1—H1A	119.3	C7—C12—H12A	120.3
C2—C1—H1A	119.3	C11—C12—H12A	120.3
C3—C2—C1	120.2 (3)	C10—C13—C14	113.7 (2)
C3—C2—H2A	119.9	C10—C13—H13A	108.8
C1—C2—H2A	119.9	C14—C13—H13A	108.8
C2—C3—C4	118.5 (3)	C10—C13—H13B	108.8
C2—C3—H3A	120.8	C14—C13—H13B	108.8
C4—C3—H3A	120.8	H13A—C13—H13B	107.7
C5—C4—C3	118.6 (3)	C13—C14—H14A	109.5
C5—C4—H4A	120.7	C13—C14—H14B	109.5
C3—C4—H4A	120.7	H14A—C14—H14B	109.5
N1—C5—C4	123.2 (2)	C13—C14—H14C	109.5
N1—C5—C6	113.8 (2)	H14A—C14—H14C	109.5
C4—C5—C6	122.9 (2)	H14B—C14—H14C	109.5
N2—C6—C5	118.6 (2)		
			
N1—Cu1—Cl1—Cu1^i^	99.07 (6)	Cu1—N1—C5—C6	8.5 (3)
Cl2—Cu1—Cl1—Cu1^i^	−103.33 (3)	C3—C4—C5—N1	0.5 (4)
Cl1^i^—Cu1—Cl1—Cu1^i^	0.0	C3—C4—C5—C6	177.0 (3)
N2—Cu1—N1—C1	174.6 (2)	C7—N2—C6—C5	178.1 (2)
Cl2—Cu1—N1—C1	−110.9 (2)	Cu1—N2—C6—C5	−8.1 (3)
Cl1—Cu1—N1—C1	−10.3 (2)	N1—C5—C6—N2	−0.3 (4)
Cl1^i^—Cu1—N1—C1	80.9 (2)	C4—C5—C6—N2	−177.0 (3)
N2—Cu1—N1—C5	−9.83 (18)	C6—N2—C7—C8	42.2 (4)
Cl2—Cu1—N1—C5	64.6 (3)	Cu1—N2—C7—C8	−130.5 (2)
Cl1—Cu1—N1—C5	165.18 (17)	C6—N2—C7—C12	−137.1 (3)
Cl1^i^—Cu1—N1—C5	−103.57 (17)	Cu1—N2—C7—C12	50.2 (3)
N1—Cu1—N2—C6	9.73 (19)	C12—C7—C8—C9	2.6 (4)
Cl2—Cu1—N2—C6	−148.34 (18)	N2—C7—C8—C9	−176.7 (2)
N1—Cu1—N2—C7	−177.3 (2)	C7—C8—C9—C10	−0.6 (4)
Cl2—Cu1—N2—C7	24.6 (2)	C8—C9—C10—C11	−0.9 (4)
C5—N1—C1—C2	−2.0 (4)	C8—C9—C10—C13	177.7 (3)
Cu1—N1—C1—C2	173.3 (2)	C9—C10—C11—C12	0.5 (4)
N1—C1—C2—C3	0.9 (4)	C13—C10—C11—C12	−178.1 (3)
C1—C2—C3—C4	1.0 (4)	C8—C7—C12—C11	−3.0 (4)
C2—C3—C4—C5	−1.7 (4)	N2—C7—C12—C11	176.3 (3)
C1—N1—C5—C4	1.3 (4)	C10—C11—C12—C7	1.5 (4)
Cu1—N1—C5—C4	−174.7 (2)	C9—C10—C13—C14	49.0 (4)
C1—N1—C5—C6	−175.4 (2)	C11—C10—C13—C14	−132.5 (3)

Symmetry codes: (i) −*x*+1, −*y*+1, −*z*+1.
